# A Five-Year-Old Slipped Capital Femoral Epiphysis Treated With Dynamic Single Screw Fixation

**DOI:** 10.7759/cureus.12992

**Published:** 2021-01-29

**Authors:** Yasuhiko Kawaguchi, Takuya Otani, Keishi Marumo, Mitsuru Saito

**Affiliations:** 1 Orthopaedic Surgery, The Jikei University School of Medicine, Tokyo, JPN

**Keywords:** slipped capital femoral epiphysis, dynamic fixation, physeal closure, growth of the proxymal femur, early onset scfe, body mass index: bmi

## Abstract

Slipped capital femoral epiphysis (SCFE) commonly occurs during puberty. Onset of SCFE at either less than 10 years old or over 16 years is defined as atypical. As in our patient, atypical onset at less than 10 years occurred in 9%, and the age of onset has been decreasing in recent years and that the probability of concomitant obesity is particularly high in young patients without obvious underlying disease or background factors. In the treatment of SCFE, preventing further slipping and permitting femoral bone growth by physeal closure is difficult, especially for young patients. We adopted ‘dynamic single screw fixation’ using SCFE short thread screw for continuous fixation without disturbing the growth of proximal femur or damaging to growth plate. Refixation was necessary once. The screw worked for 7 years 4 months while physeal closure was avoided. At the 10-year follow-up, her growth had stopped. She had no problem clinically, no increase in the posterior sloping angle (PSA), and no obvious growth disturbance of the femur.

## Introduction

Slipped capital femoral epiphysis (SCFE) commonly occurs during puberty when there is rapid physical growth. According to an international multicenter study, the mean age of onset is 13.6 ± 1.7 for boys and 12.2 ± 1.3 for girls [1]. The reasons for this include increased mechanical stress due to obesity and sports activities, and endocrinological factors. In the same report, the age of onset was divided into three categories: onset at less than 10 years, onset from 10 to 16 years, and onset at over 16 years. Onset at either less than 10 years or over 16 years was defined as atypical. Most of the atypical cases were caused by endocrinological disease such as hypothyroidism [2-3], metabolic disease such as renal osteodystrophy [2, 4], or following radiotherapy or chemotherapy [5-6]. According to a report by Loder, atypical onset less than 10 years occurred in 40 of 433 subjects [1]. Lehmann et al. noted that the age of onset has been decreasing in recent years and considered whether earlier maturation might be the cause [5]. Another report indicates, however, that if earlier maturation is indeed the cause, the number of young patients should be higher, and suggests the possibilities of multifactorial causes [7]. Reports indicate that the probability of concomitant obesity is particularly high in young patients without obvious underlying disease or background factors [8-9].

Here, we report a case of very early onset SCFE, occurring at the age of 5 years, which was treated with continuous screw fixation for a period of 7 years 4 months while avoiding early physeal closure. Follow-up was conducted for a period of 10 years after treatment, including the period after the end of growth.

## Case presentation

**Figure 1:**
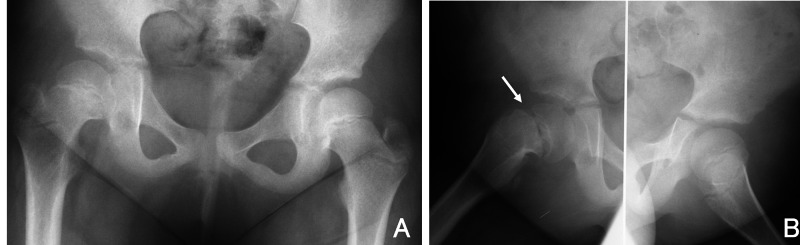
Preoperative imaging (5 years 3 months old). (A) Anteroposterior and (B) Rauenstein radiographs of the bilateral hip show a posterior-inferior slip of the femoral epiphysis of the right hip (arrow).

**Figure 2:**
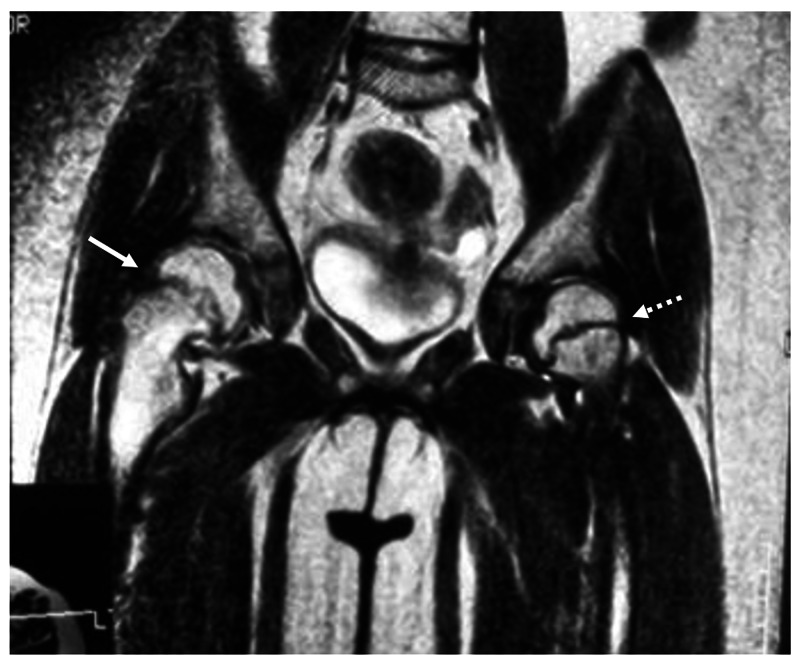
Preoperative MRI T2 image. A coronal MRI image shows bone marrow edema surrounding the right physis (arrow), but there were no findings suggesting pre-slip on the left (arrow of the thick dotted line).

A girl aged 5 years 3 months presented with a four-month history of pain around the right knee joint and a limp. Both she and her mother were obese. On initial examination, her height was 120 cm, weight 45 kg, and body mass index (BMI) 30.8 kg/m2. She showed an obvious limp and the range of motion in the right/left hips was flexion, 120°/130°; abduction, 35°/45°; internal rotation, −20°/30°; and external rotation, 75°/75°. Drehmann’s sign was positive on the right. She did not have previous history of trauma. No abnormalities or signs of an endocrine disorder were observed in the laboratory tests. Plain X-rays showed widening and irregularity of the physis of the right proximal femur, and the posterior sloping angle (PSA) was 35° on the right and 3° on the left (Figure 1). MRI showed obvious bone marrow edema surrounding the right physis but there were no findings suggesting pre-slip on the left (Figure 2). Despite the very young age of onset [10], she was diagnosed with stable SCFE.

Surgery

**Figure 3:**
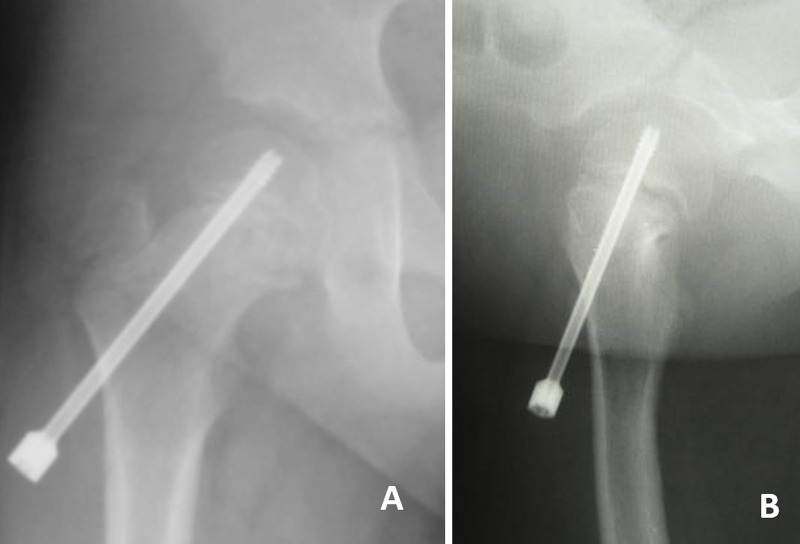
Postoperative imaging (first surgery). (A) Anteroposterior and (B) Rauenstein radiographs of the right hip following in situ dynamic single screw fixation.

In situ dynamic single screw fixation was performed under general anesthesia. We used a short thread, cannulated, cancellous screw of titanium alloy (SCFE screw; MEIRA Corp., Nagoya, Japan). The screw was inserted from the anterolateral direction perpendicularly to the physis; the entire thread was advanced into the epiphysis so that the thread did not bridge the physis, and the screw head projected 25 mm laterally from the cortex (Figure 3). Prophylactic fixation on the contralateral side was not performed.

**Figure 4:**
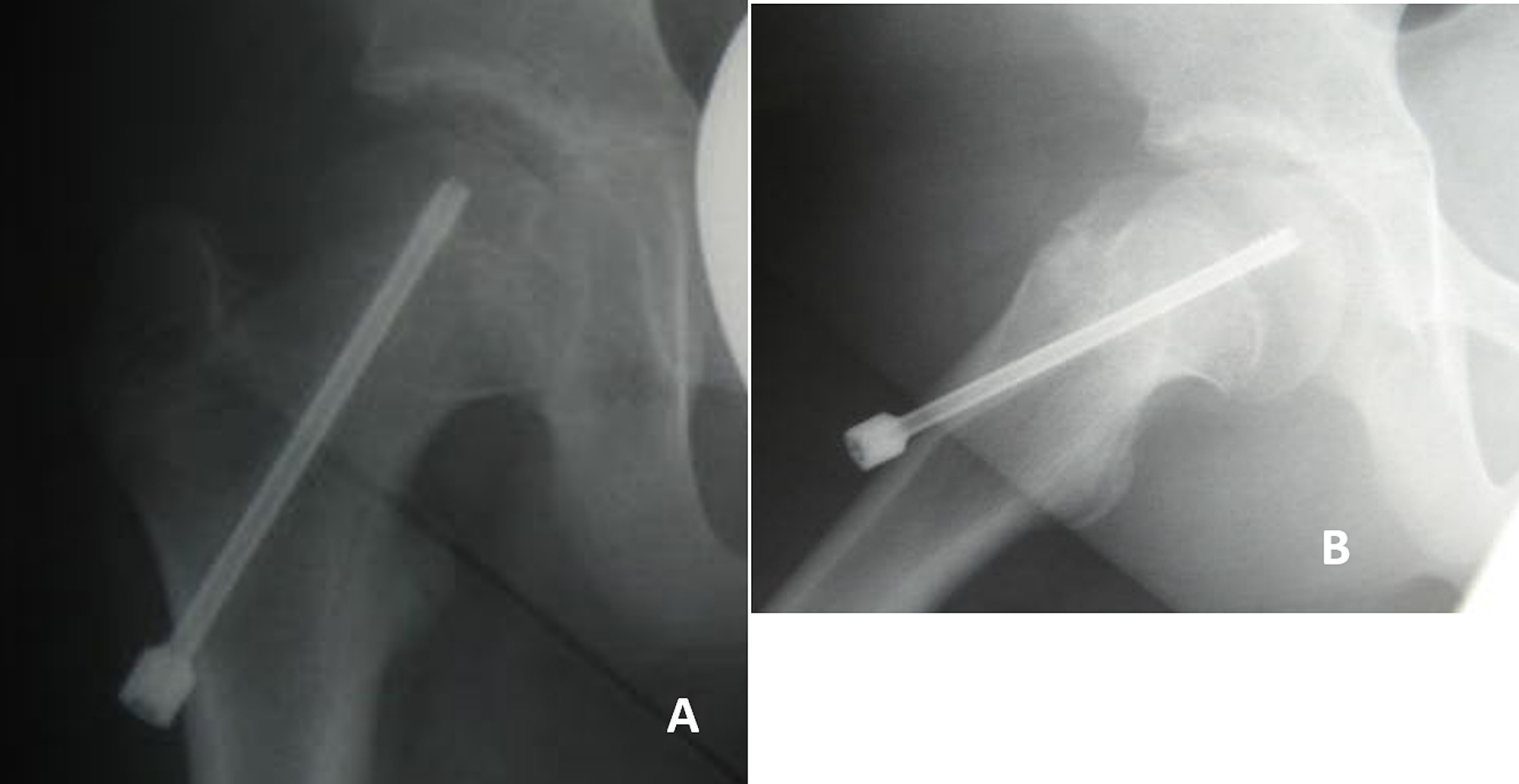
Postoperative imaging (3 years after first surgery). (A) Anteroposterior and (B) Rauenstein radiographs of the right hip, the lateral end of the screw was gradually incorporated into the femur and approached the cortex.

The hip pain rapidly disappeared postoperatively and full weight bearing was permitted after four months. Follow-up X-rays showed that the physis on the affected side regained its smooth appearance and physeal closure did not occur. In addition to favorable growth of the proximal femur, the lateral end of the screw was gradually incorporated into the femur and approached the cortex (Figure 4). Surgery was then performed to replace the screw 3 years 4 months after the initial surgery at the age of 8 years 7 months (height, 144 cm; weight, 73 kg; BMI, 35.2).

Second surgery

**Figure 5:**
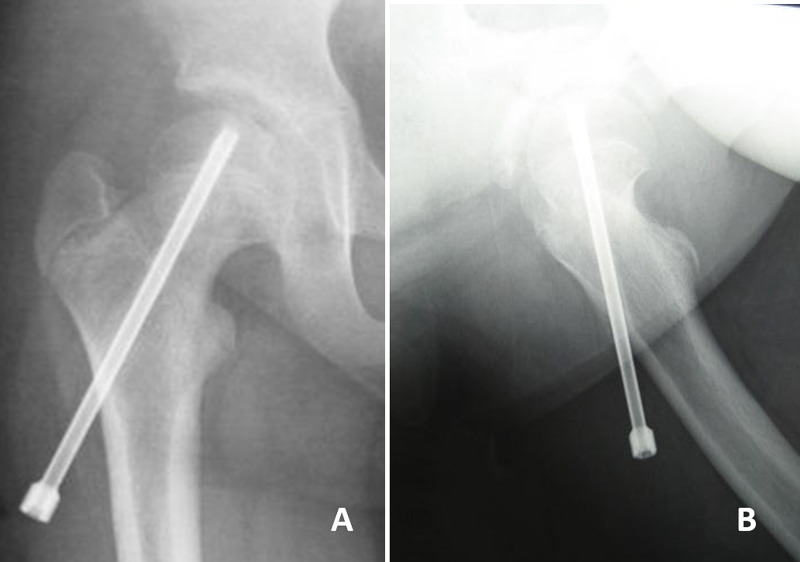
Postoperative imaging (second operation; 3 years 4 months after first surgery). (A) Anteroposterior and (B) Rauenstein radiographs of the right hip; we replaced the other longer screw through the same screw hole.

We made a 1-cm incision in the original scar, removed the screw that was passed through a guidewire, and replaced the other longer screw. The same kind of short thread screw was used; the diameter and length of the thread were 6.5 and 7 mm, respectively, the diameter of the smooth portion was 5.0 mm, and the total length was 120 mm. Again, the thread was completely inserted into the epiphysis, and the screw head projected 30 mm laterally (Figure 5).

**Figure 6:**
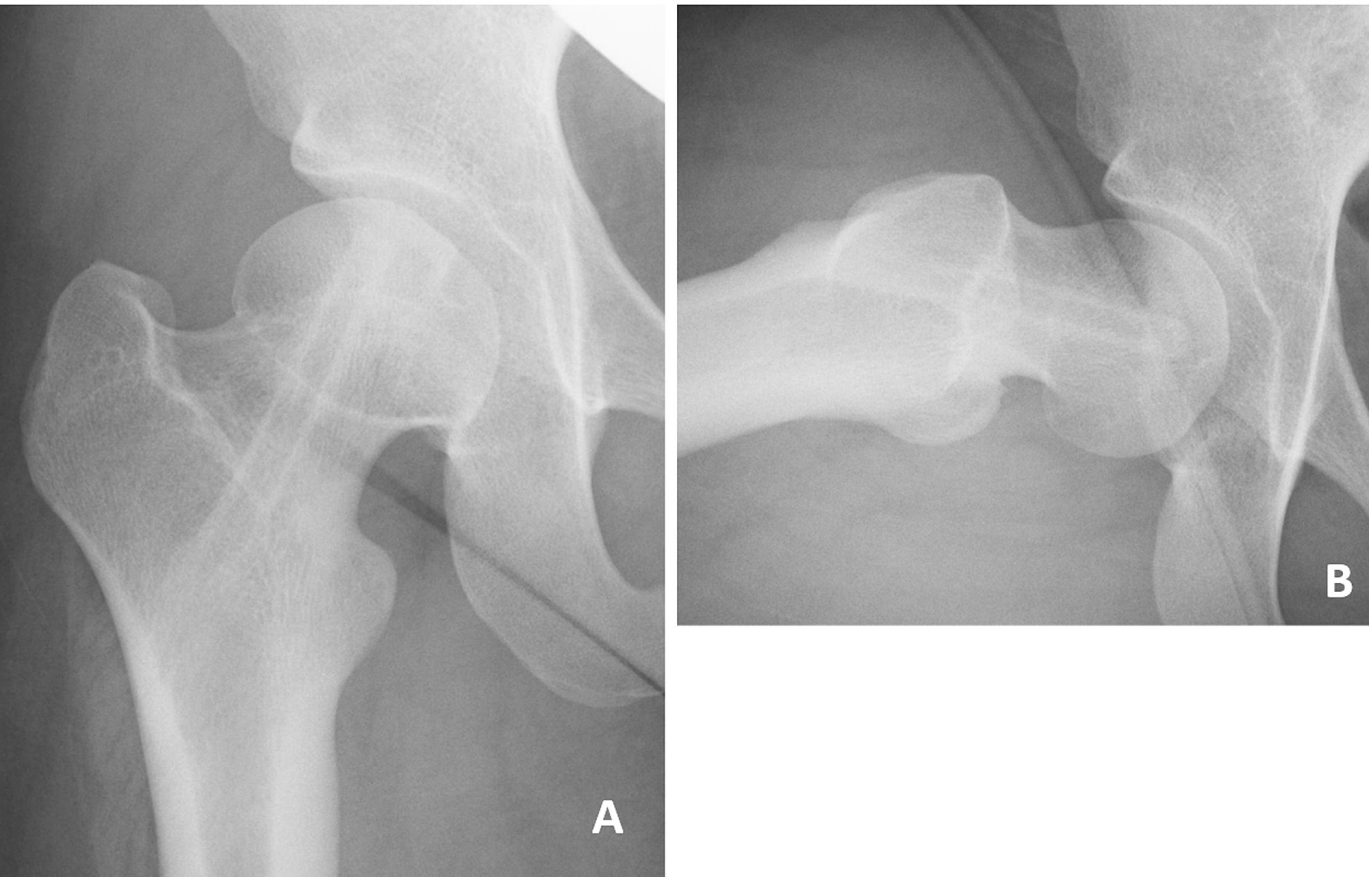
Imaging at last follow up (15 years old). (A) Anteroposterior and (B) axial radiographs of the right hip, at final follow up (end of her bone growth; 10 years after the first surgery).

Her age at menarche was 11 years. As physeal closure was observed at 12 years nearly simultaneously for both sides, the screw was removed at the age of 12 years 7 months (7 years 4 months after the initial surgery). The final follow-up was conducted at age 15 (10 years after the initial surgery); her height was 154 cm, weight 83 kg, and BMI 35. There was no pain, no limp, and she could participate in sports. Range of motion in the right/left hip joints was: flexion, 110°/110°; abduction, 30°/40°; internal rotation, 5°/20°; and external rotation, 65/45°, respectively. Drehmann’s sign was not observed in the right hip and there were no signs or symptoms of femoroacetabular impingement (Figure 6).

## Discussion

The SCFE occurs when the capital femoral epiphysis displaces through the fragile physis from the metaphysis. Previous literature has identified obesity as the main risk factor for SCFE; overweight contributes to increased sheer stress across the physis. With regard to the relationship between the onset of SCFE and BMI, Poussa et al. reported that BMI could be a useful tool for evaluating the risk of SCFE [9], and Manoff et al. reported that 81.1% of 106 patients had a BMI that exceeded the 95th percentile [7]. In addition, Bhatia et al. reported that BMI was higher in patients with bilateral occurrence than those with unilateral occurrence [8]. In young SCFE patients without obvious underlying disease or background factors, reports indicate that the probability of concomitant obesity is particularly high [10]. Our patient’s BMI was also high (30.8 kg/m2) at the initial examination and increased with growth. We believe that increased mechanical stress on the physis caused by obesity was an important factor because she had no endocrinological abnormalities and had not taken part in sports activities.

She was considered a borderline case with respect to the standard treatment based on the PSA, which is usually in situ fixation and trochanteric osteotomy. Because of her young age, we expected that favorable remodeling would occur and we therefore performed a less invasive in situ fixation. In addition, we used a dynamic method [11] to prevent early physeal closure.

Treatment outcomes of single screw in situ fixation in mild to moderate stable-type SCFE are favorable. Aronson and Carlson reported that the proportions with excellent or good outcomes were 95% for mild slips and 91% for moderate slips [12], and it has become a common treatment worldwide in recent years. However, sufficient consideration needs to be given to early physeal closure when treating young patients like the present case. The physis becomes mechanically unstable in SCFE and some believe that it is better to treat the condition by ensuring physeal closure. However, Ward et al. reported physeal closure after a mean of 13 months (2-34 months) following single screw in situ fixation, and mentioned that early physeal closure caused a leg length discrepancy and a restricted range of movement [13]. Kim et al. investigated 85 subjects who underwent single screw fixation and found a leg length discrepancy ≥1 cm in 48 subjects (56%) and ≥2 cm in 10 subjects (12%) 6 years postoperatively [14]. Segal et al. also observed shortened articulo-trochanteric distance (ATD) compared with the unaffected limb who underwent in situ pinning; they stated that precautions need to be taken to prevent early physeal closure [15]. Therefore, preventing further slipping and permitting femoral bone growth while preventing physeal closure is difficult to achieve in the treatment of SCFE especially for young patients.

So, we used the dynamic method reported by Kumm et al. [11], by using a short thread screw; the thread does not bridge the physis and the entire thread is placed within the epiphysis. The screw head projects laterally, and the screw may be incorporated into the femur as the femoral neck grows. The screw is inserted perpendicular to the physis. All of these methods play important roles in permitting femoral neck growth and preventing early physeal closure. Kumm et al. reported the outcomes of this method for preventative fixation of the unaffected side in SCFE patients: no cases of early physeal closure were noted in 34 hips after a mean follow-up of 5.4 years. They then reported the use of the dynamic method to treat 29 mild SCFE (slip angle ˂ 30°) in 2001 [16], and there were no complications such as slip progression, avascular necrosis, or chondrolysis. Early physeal closure occurred 6 months earlier than in the contralateral side in 5 of 20 unilateral SCFE, and the difference in leg length in these subjects ranged from 0 to 10 mm (mean 3 mm).

Although there are favorable reports described by Kumm et al., to the best of our knowledge, there are no other follow-up studies of the dynamic method with the exception of a report by Guzzanti et al. [17] who used a fixation that was similar to the dynamic method. They used a modified single cannulated screw to treat 10 hips in skeletally immature patients and reported that premature physeal closure was prevented. There are also reports of a modified method by Hansson [18] and Hägglund et al. [19] at the same institution using a hook-pin. Hansson reported that 27 of 37 hips (73%) showed no tendency towards premature physeal closure.

Age range of the 25 subjects in the report by Kumm et al. was 9.4-16.1 years [16], and that of the 10 subjects in the report by Guzzannti et al. was 10.6-12.6 years [17]. There are no reports regarding treatment in very young subjects. We believe that the age of 5.4 years in the present case is the youngest age for which the dynamic single screw fixation was used. And we used the short thread SCFE screw, which has a diameter and length of the thread of 5.5 and 7 mm, respectively; the diameter of the smooth portion was 4 mm, and the total length was 90 mm. The PSA was 27° on the right and 14° on the left. At the final follow-up, α angles were 70° on the right and 40°on the left. The ATD was 56% and the femoral neck length was 95% of the value of the unaffected side (Figure 6). The final difference in leg length was 6 mm.

## Conclusions

We reported the treatment of a female child aged 5 years with stable SCFE by using a dynamic single screw fixation method for total duration 7 years 4 months, with one screw replacement. Premature physeal closure was avoided and the final difference in leg length was 6 mm. We believe that the described method is extremely useful for treating early-onset SCFE over a long period of time until physeal closure is achieved.
